# Comparison of short-segment monoaxial and polyaxial pedicle screw fixation combined with intermediate screws in traumatic thoracolumbar fractures: a finite element study and clinical radiographic review

**DOI:** 10.6061/clinics/2017(10)04

**Published:** 2017-10

**Authors:** Hongwei Wang, Yiwen Zhao, Zhongjun Mo, Jianda Han, Yu Chen, Hailong Yu, Qi Wang, Jun Liu, Changqing Li, Yue Zhou, Liangbi Xiang

**Affiliations:** I Department of Orthopedics, General Hospital of Shenyang Military Area Command of Chinese PLA, Shenyang, Liaoning, 110016, China; II State Key Laboratory of Robotics, Shenyang Institute of Automation, Chinese Academy of Science, Shenyang, Liaoning, 110016, China; III National Research Center for Rehabilitation Aids, Beijing, 100176, China; IV Department of Orthopedics, Xinqiao Hospital, the Third Military Medical University, Chongqing, 400037, China

**Keywords:** Biomechanics, Finite Element Analysis, Spine Fracture, Monoaxial Pedicle Screw, Polyaxial Pedicle Screw

## Abstract

**OBJECTIVES:**

No studies have compared monoaxial and polyaxial pedicle screws with regard to the von Mises stress of the instrumentation, intradiscal pressures of the adjacent segment and adjacent segment degeneration.

**METHODS:**

Short-segment monoaxial/polyaxial pedicle screw fixation techniques were compared using finite element methods, and the redistributed T11-L1 segment range of motion, largest maximal von Mises stress of the instrumentation, and intradiscal pressures of the adjacent segment under displacement loading were evaluated. Radiographic results of 230 patients with traumatic thoracolumbar fractures treated with these fixations were reviewed, and the sagittal Cobb’s angle, vertebral body angle, anterior vertebral body height of the fractured vertebrae and adjacent segment degeneration were calculated and evaluated.

**RESULTS:**

The largest maximal values of the von Mises stress were 376.8 MPa for the pedicle screws in the short-segment monoaxial pedicle screw fixation model and 439.9 MPa for the rods in the intermediate monoaxial pedicle screw fixation model. The maximal intradiscal pressures of the upper adjacent segments were all greater than those of the lower adjacent segments. The maximal intradiscal pressures of the monoaxial pedicle screw fixation model were larger than those in the corresponding segments of the normal model. The radiographic results at the final follow-up evaluation showed that the mean loss of correction of the sagittal Cobb’s angle, vertebral body angle and anterior vertebral body height were smallest in the intermediate monoaxial pedicle screw fixation group. Adjacent segment degeneration was less likely to be observed in the intermediate polyaxial pedicle screw fixation group but more likely to be observed in the intermediate monoaxial pedicle screw fixation group.

**CONCLUSION:**

Smaller von Mises stress in the pedicle screws and lower intradiscal pressure in the adjacent segment were observed in the polyaxial screw model than in the monoaxial pedicle screw fixation spine models. Fracture-level fixation could significantly correct kyphosis and reduce correction loss, and adjacent segment degeneration was less likely to be observed in the intermediate polyaxial pedicle screw fixation group.

## INTRODUCTION

The posterior transpedicular pedicle screw fixation technique has been widely adopted for the management of an unstable spine, mainly due to trauma, and approximately 10-20% of such injuries are burst fractures 1,2. Despite the advantages of a short-segment spinal instrumentation approach, such as improved correction of the spinal deformity, this procedure has also been associated with instrumentation failure, such as screw loosening, screw breakage and correction loss in some cases 3-7. Reinforcement of fixation at the fracture level can help to improve kyphosis correction and biomechanical stability 8-14. As a result, improved design and implantation techniques of pedicle screws have reduced the rate of pedicle screw and rod breakage and facilitated efficient application of the connecting rod without undue stress on the construct 15-19. Compared to a monoaxial screw design, the compression and bending strength at the polyaxial head is reduced due to its specific structural design 17,18, but no studies have compared monoaxial and polyaxial pedicle screws with regard to the range of motion (ROM), von Mises stress (VMS) of the internal fixation devices, intradiscal pressures (IDPs) of the adjacent segment, or adjacent segment degeneration (ASD) using radiographic reviews.

In the current study, the biomechanical characteristics of fixation techniques (SM4+2/SP4+2: short-segment monoaxial/polyaxial pedicle screw fixation; IM6+2/IP6+2: intermediate monoaxial/polyaxial pedicle screw fixation) were compared, using finite element methods, with regard to the redistributed ROM, the VMS of internal fixation devices, and the IDPs of the adjacent segment under displacement loading. Two hundred thirty patients presenting with traumatic thoracolumbar fractures and treated with these types of fixation techniques were reviewed, and ASD was evaluated.

## MATERIALS AND METHODS

### Biomechanical Study

A finite element model including seven vertebrae and six discs between T9 and L3 of the spine was reconstructed and analyzed using finite element analysis software 14,. The fixation models are described as SM4+2, IM6+2, SP4+2 and IP6+2. In the current study, SM4+2/SP4+2 is defined as short-segment monoaxial/polyaxial pedicle screw fixation, and IM6+2/IP6+2 is defined as intermediate monoaxial/polyaxial pedicle screw fixation. Surface-to-surface contact was defined between articulation facets. The diameter and length of the screws were 6 mm and 45 mm, respectively. The pedicle screws in the current study included monoaxial and polyaxial pedicle screws. The constraint was defined between polyaxial pedicle screw heads and shafts. However, a load limitation was defined. Surface-to-surface contact was defined between polyaxial pedicle screw heads and shafts. The screw tilt (the maximal deviation of the long axis of the screw away from perpendicular to the longitudinal rod) was 25°, and the static torque was 8 Nm, which indicated that the polyaxial pedicle screw heads will move relative to the shafts when the torque between the heads and shafts reaches 8 Nm. In our previous study 14, which used only monoaxial pedicle screws, we only applied a pure moment of 10 Nm combined with a precompressive load of 150 N to the fixation models 23-25 before measuring the ROM of the T11-L1 segment. In the current study, we measured the ROM of the intact spine model of T9-L3 under flexion, extension, left/right lateral bending and left/right axial rotation and then applied ROM displacement loading to the four fixation models. Our previous study showed that the calculation model presented in this paper is rational 14, but the pattern of mechanical load presented here is different from the previous study. The redistributed ROM of the T11-L1 segment, the largest maximal von Mises stress (LMS) of the instrumentation, and the IDPs of the adjacent segment under displacement loading were evaluated.

### Radiographic Review

Two hundred thirty patients presenting with a single traumatic thoracolumbar fracture with a fractured vertebral body between T11 and L3 underwent surgery in our hospitals using posterior short segment fixation techniques. The inclusion criteria consisted of a traumatic T11-L3 fracture, a type A fracture according to the AO classification 26, the absence of neurologic deficits, age of 18 to 50 years, a time interval between trauma and surgery of less than 15 days, and a follow-up period longer than 12 months. The exclusion criteria were a pathologic or osteoporotic fracture, lumbar degenerative diseases, or a history of thoracic or lumbar surgery. The SP4+2, IP6+2, SM4+2 and IM6+2 groups included 27, 50, 53 and 100 patients, respectively (Table 1). The sagittal Cobb’s angle (SCA), vertebral body angle (VBA) and anterior vertebral body height (AVBH) of the fractured vertebrae were calculated as described previously 19,27,28. The correction loss refers to the loss of the SCA, VBA and AVBH during the follow-up period compared to the immediate postoperative results. ASD was defined, according to a previous study 28, as one or more of the following conditions: 1) a preoperative disc height with more than 20% correction loss, 2) retrolisthesis or anterolisthesis of more than 3 mm, or 3) an osteophyte greater than 3 mm. The ethics committee of Xinqiao Hospital approved the procedures (registry number: 20100030), and written informed consent was obtained from patients.

### Statistical Analysis

We used SPSS 15.0 software (SPSS Inc., Illinois, USA) to perform all statistical analyses, and a value of *p*<0.05 was considered significant (two-tailed). Independent samples t-tests and one-way ANOVA were used to compare group means. The chi-square test was conducted to assess frequency data when appropriate.

## RESULTS

### Biomechanical Study

**Figure 1:**
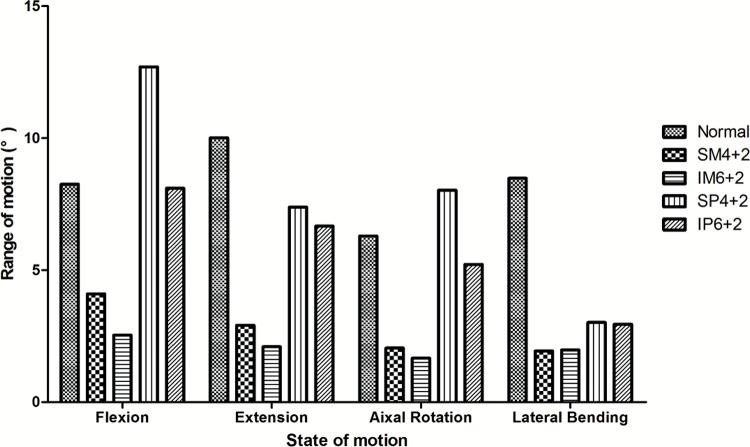
Range of motion (ROM). **SM4+2/SP4+2**: short-segment monoaxial/polyaxial pedicle screw fixation; **IM6+2/IP6+2**: intermediate monoaxial/polyaxial pedicle screw fixation.

The SM4+2, IM6+2 and IP6+2 fixation models presented a decreased ROM compared to the intact normal spine model in all states of motion, and these results are illustrated in Figure 1. The redistributed ROM was smallest in the IM6+2 fixation model for flexion, extension and axial rotation, and the redistributed ROM was largest in the SP4+2 fixation model, especially for flexion, for which the ROM was larger than in the intact spine model.

**Figure 2:**
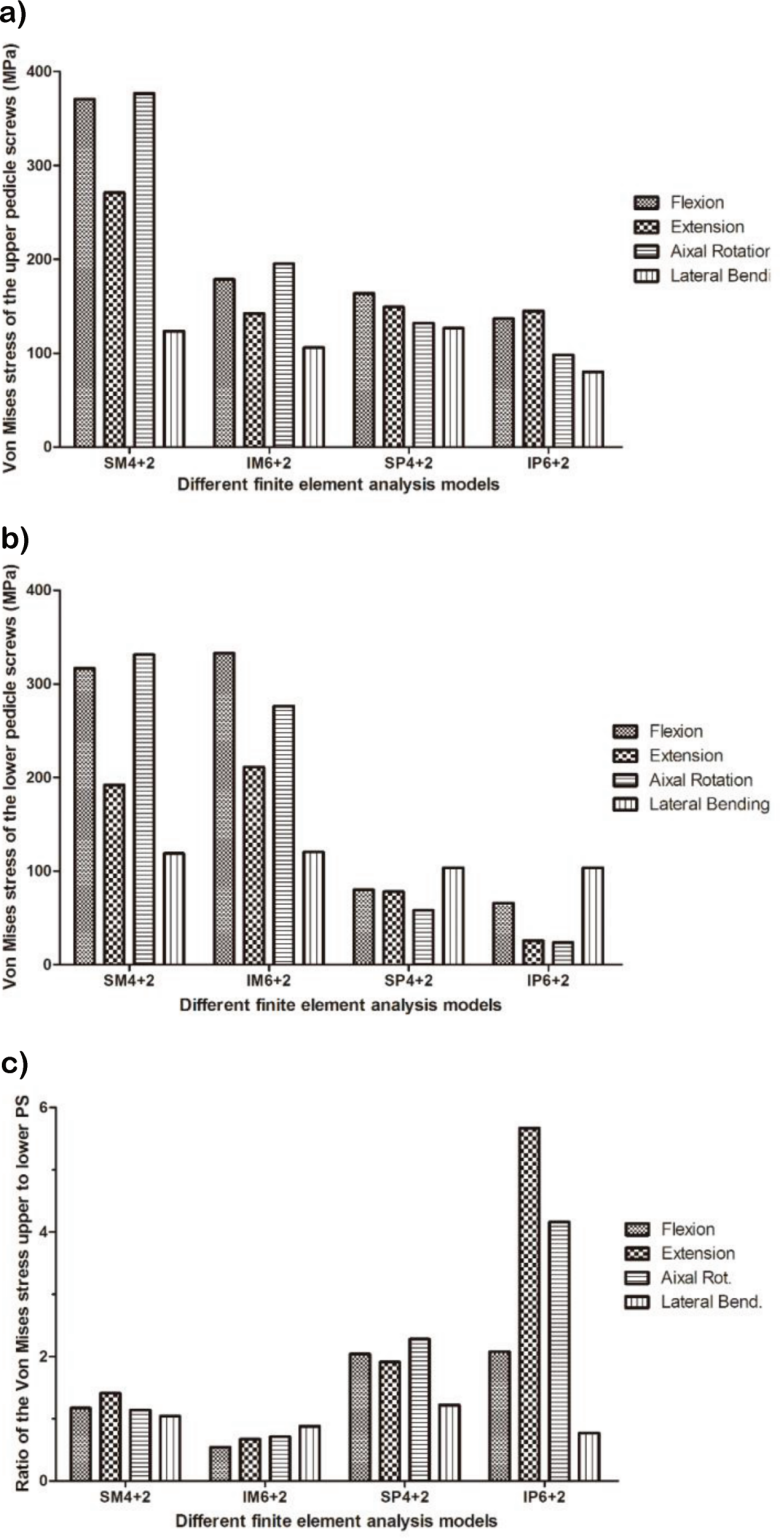
von Mises stress of the pedicle screws. A. Upper pedicle screws. B. Lower pedicle screws. C. Ratio of von Mises stress of the upper to the lower pedicle screws. **SM4+2/SP4+2**: short-segment monoaxial/polyaxial pedicle screw fixation; **IM6+2/IP6+2**: intermediate monoaxial/polyaxial pedicle screw fixation.

**Figure 3:**
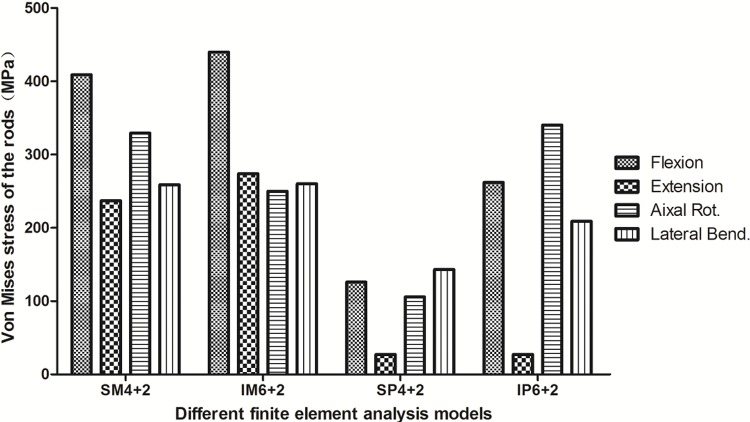
von Mises stress of the rods. **SM4+2/SP4+2**: short-segment monoaxial/polyaxial pedicle screw fixation; **IM6+2/IP6+2**: intermediate monoaxial/polyaxial pedicle screw fixation.

The LMS of the pedicle screws was observed in flexion and axial rotation in the monoaxial pedicle screw fixation models (MPSF) and in flexion and extension in the polyaxial pedicle screw fixation models (PPSF). The upper pedicle screw presented the LMS in the SM4+2, SP4+2 and IP6+2 models, and the lower pedicle screw presented the LMS in the IM6+2 model (Figure 2). The LMS values of the pedicle screws were 376.8 MPa, 332.8 MPa, 163.8 MPa and 145.0 MPa for the SM4+2, IM6+2, SP4+2, and IP6+2 models, respectively. The LMS of the rods was observed during flexion in the MPSF models and during lateral bending and rotation in the PPSF models (Figure 3). The LMS values of the rods were 409.9 MPa, 439.9 MPa, 143.1 MPa and 340.3 MPa for the SM4+2, IM6+2, SP4+2, and IP6+2 models, respectively. The results of stress distribution within the instruments demonstrated that the largest stress occurred at the pedicle screw root during flexion.

**Figure 4:**
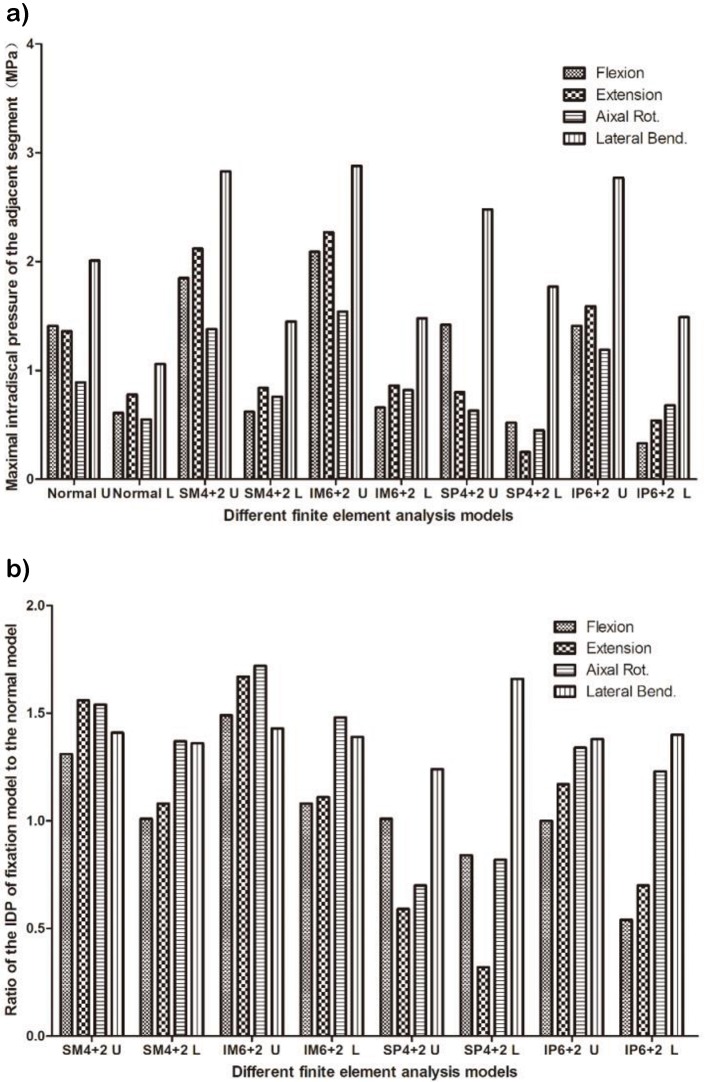
Intradiscal pressure of the adjacent segment. A. Maximal intradiscal pressure of the adjacent segment. B. Ratio of the IDP of the fixation model to that of the normal model. **SM4+2/SP4+2**: short-segment monoaxial/polyaxial pedicle screw fixation; **IM6+2/IP6+2**: intermediate monoaxial/polyaxial pedicle screw fixation; **U**: upper adjacent segment; **L**: lower adjacent segment; **IDP**: intradiscal pressure.

Maximal IDPs of the adjacent segment were observed during lateral bending. The maximal IDPs of the upper adjacent segments were all larger than those of the lower adjacent segments. In the MPSF models, the maximal IDPs were larger than those in the corresponding segment of the normal model. In the PPSF models, some IDPs of the adjacent segments were less than those of the normal model (Figure 4).

### Radiographic Review

**Figure 5:**
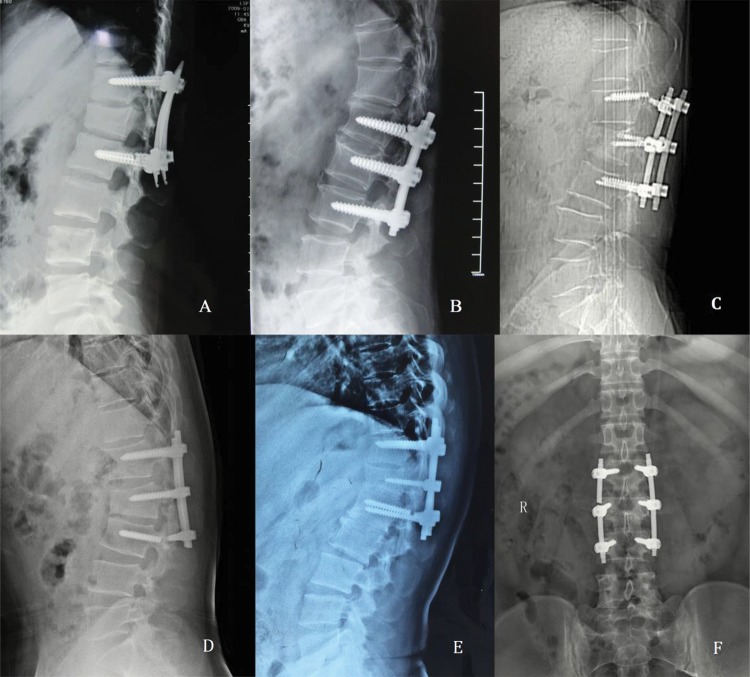
Illustrative cases presented with pedicle screw and rod breakage. A. Pedicle screw breakage occurred 14 months after surgery in a 14-year-old girl with an L1 fracture. B. Pedicle screw breakage occurred 10 months after surgery in a 47-year-old man with an L2 fracture. C. Pedicle screw breakage occurred 18 months after surgery in a 39-year-old woman with a T12 fracture. D. Pedicle screw breakage occurred 8 months after surgery in a 35-year-old man with an L2 fracture. E. Pedicle screw breakage occurred 12 months after surgery in a 45-year-old man with a T12 fracture. F. Rod breakage occurred 24 months after surgery in a 27-year-old man with an L3 fracture.

The postoperative SCA, VBA and AVBH were significantly improved compared to the preoperative values in all four groups. The postoperative SCA in the SP4+2 group and the AVBH in the IM6+2 group were significantly larger than the preoperative values. The final follow-up evaluation showed that the mean loss of correction of the SCA, VBA and AVBH were smallest in the IM6+2 group (Table 2). In the SM4+2 group, screw loosening occurred in four patients (4/53, 7.5%). In the IM6+2 group, breakage of pedicle screws (4 patients) or rods (1 patient) was observed in five patients (5/100, 5.0%). In the SP4+2 group, two patients (2/27, 7.4%) presented with pedicle screws breakage, and one patient (1/27, 3.7%) presented with screw loosening. No patients in the IP6+2 group presented with pedicle screw breakage, rod breakage, or screw loosening. Illustrative cases with pedicle screw or rod breakage are presented in Figure 5. Two patients (1/53, 1.9%) in the SM4+2 group, two patients (3/100, 3.0%) in the IM6+2 group, and no patients in the SP4+2 and IP6+2 groups presented with ASD.

## DISCUSSION

Reinforcement with fixation at the fracture level can help to improve kyphosis correction and biomechanical stability 8-14. However, no studies have compared monoaxial and polyaxial pedicle screws with regard to the VMS of the instrumentation and the IDP of the adjacent segment. Our previous study suggested that the intermediate screw fixation technique can significantly increase the stability of the spine in both the MPSF and PPSF groups. However, the MPSF group exhibited more stability in flexion and extension than the PPSF group 13. Because we previously could not measure the VMS of the instrumentation or the IDP of the adjacent segment 13, we performed a finite analysis study to determine the biomechanical comparisons between monoaxial or polyaxial pedicle screws in thoracolumbar fractures. Fixation models, including the SM4+2, IM6+2 and IP6+2 models, exhibited a lower ROM than the intact normal spine model. The ROM was smallest in the IM6+2 model. In contrast, the ROM was largest in the SP4+2 model, especially during flexion, during which the ROM was larger than that in the intact spine model. The biomechanical results were consistent with our radiological results. The postoperative SCA, VBA and AVBH were significantly improved compared to the preoperative values in all four groups. The final follow-up evaluations showed that the mean loss of correction of the SCA, VBA and AVBH was smallest in the IM6+2 group and largest in the SP4+2 group. This phenomenon can be explained because polyaxial pedicle screws heads are vulnerable to fatigue failure; the region between the screw head and shaft were found to fail first in many biomechanical studies 29-31. The failure may then result in loss of correction of the SCA, VBA and height of the vertebral bodies.

The LMS of the upper pedicle screw was larger than that of the lower pedicle screw in the SM4+2, SP4+2 and IP6+2 models. The LMS values of the instrumentation in the MPSF group were larger than those in the PPSF models. These results may suggest that the PPSF technique can decrease the VMS of the instrumentation. Upon suspecting that a pedicle screw is broken, the upper pedicle screw must be the focus of attention for patients subjected to SM4+2, SP4+2 and IP6+2 fixation techniques, and the lower pedicle screw is the focus for the IM6+2 fixation technique. Clinically, 12 patients presented with fixation failure, including instrument breakage and screw loosening. Six patients presented with pedicle screw breakage, all of which occurred at the root of the pedicle screw because the results of stress distribution within the instruments demonstrated that the largest stress occurred at the pedicle screw root.

In the SP4+2 group, pedicle screw breakage was observed in 2 patients (one upper pedicle screw, one lower pedicle screw), and screw loosening occurred in 1 patient (upper pedicle screw). In the SM 4+2 group, screw loosening occurred in 4 patients (three upper pedicle screws, one lower pedicle screw). We suggest that the phenomenon may be related to the fact that the upper pedicle screw presented the LMS in the SP4+2 and SM4+2 groups, and the redistributed ROM was largest in these two fixation models. The rate of screw loosening in the SM 4+2 group (7.5%) was significantly higher than in the SP 4+2 group (3.7%). It is difficult to precisely align the side opening of two monoaxial pedicle screw heads for insertion of the rod, and small discrepancies in the fixation of monoaxial pedicle screws may increase the rotational forces and intervertebral translational forces with negative effects at the screw-to-vertebral-body interface, which may lead to screw cut-out or loosening 29,32,33. In the IM6+2 group, pedicle screw breakage (two upper pedicle screws, two lower pedicle screw) and rod (1 patient) was observed in 5 patients. According to a previous study 34, the fatigue stress, yield stress, and ultimate stress (fracture stress) were 550 MPa, 869 MPa, 924 MPa, respectively. In the current study, the LMS values were 376.8 MPa in all pedicle screws and 439.9 MPa in all rods. We suggest that this phenomenon may be related the patients all being manual workers who often bend over, and the lordosis angles of the rods were so large that the stress of the pedicle screws increased. The LMS value of the rods was largest in the IM6+2 group. In the SP6+2 group, no patients presented with pedicle screw breakage, rod breakage, or screw loosening. By comparison, additional intermediate polyaxial pedicle screws may result in a stiffer construct than the normal spine model and less VMS of the pedicle screws than in the MPSF models. The LMS of the pedicle screws was observed in flexion and axial rotation in the MPSF models and in flexion and extension in the PPSF models; the LMS of the rods was observed in flexion in the MPSF models but in lateral bending and rotation in the PPSF models. Therefore, we suggested that patients should be prohibited from bending over, rotating abruptly or bearing heavy weight on their back during the early postoperative stage.

In the current study, the maximal IDPs of the adjacent segment were observed during lateral bending. The maximal IDPs of the upper adjacent segments were all larger than those of the lower adjacent segments in the MPSF models. These results were consistent with those of previous studies 35-39. Rahm et al. 35 noted that upper ASD can develop more easily than lower ASD after lumbar fusion with instrumentation. Chen et al. 37 observed that the upper disc adjacent to the interbody fusion presented with a larger stress than the lower disc. In the MPSF models, the maximal IDPs were larger than in the corresponding segments of the normal model. These results were consistent with previous studies that noted that fusion accelerates ASD compared to the natural history 40-42, although Radcliff KE 43 reported that spinal operations are associated with ASD. However, whether such operations accelerate the natural history of ASD remains unknown. In the current PPSF model, some IDPs of the adjacent segments were lower than those of the normal model. Additional intermediate polyaxial pedicle screws may result in lower IDPs of adjacent segments than in MPSF models. Our radiological results showed that ASD was less likely to be observed in the IP6+2 group but more likely to be observed in the IM6+2 group.

This study has many limitations. First, the number of patients included in the study was small, and this was a retrospective study. Second, a selection bias may exist because this study included patients referred to our teaching hospitals. Third, it is necessary to discuss several factors, including different patient conditions, muscle forces, ribs, and the length and diameter of pedicle screws, for a more clinically feasible conclusion because these factors can influence finite element analysis results. Fourth, the stress of the screw-to-vertebral-body interface was not analyzed in the current study; we will conduct further research specifically considering spinal models with different bone quality in the near future.

The use of additional intermediate monoaxial pedicle screws may result in a stiffer construct and reduced VMS of the pedicle screws than in SPSF models. In comparison, use of additional intermediate polyaxial pedicle screws may result in a stiffer construct than the normal spine model and lower VMS of the pedicle screws and IDPs of the adjacent segment than MPSF models. Fracture-level fixation could significantly correct kyphosis and reduce correction loss, and ASD was less likely to occur in the IP6+2 group. Our results provided a theoretical basis for the treatment of spinal fractures using minimally invasive intermediate polyaxial pedicle screw fixation.

## AUTHOR CONTRIBUTIONS

Wang H, Zhao Y and Mo Z conceived of the study, collected the data, participated in the data analysis, drafted the manuscript and performed the statistical analysis. Han J conceived the study and participated in its design, coordination and drafting. Chen Y, Yu H, Wang Q, Liu J and Li C participated in the data analysis and interpretation. Zhou Y and Xiang L participated in the review, coordination and drafting of the manuscript and contributed to the analysis via constructive discussions.

## Figures and Tables

**Table 1:** Preoperative demographic and clinical data [Mean±SD].

Variable	SM4+2	SP4+2	IM6+2	IP6+2
Patients (n)	53	27	100	50
Mean age (years)	37.4±9.3	36.0±10.3	38.7±8.3	37.5±9.5
Sex ratio (M/F)	36/17	19/8	65/35	36/14
AO fracture classification (A1/A2/A3)	33/9/11	15/5/7	55/20/25	27/9/14
Time interval from injury to operation (days)	6.5±2.9	6.7±2.9	6.9±3.1	6.6±3.0
Mean ISS	13.7±7.1	12.7±6.5	13.8±7.1	13.6±7.2
Follow-up (months)	22.4±8.1	21.3±10.5	22.4±8.0	24.9±5.9

**SM4+2/SP4+2**: short-segment monoaxial/polyaxial pedicle screw fixation; **IM6+2/IP6+2**: intermediate monoaxial/polyaxial pedicle screw fixation; **ISS**: injury severity score.

**Table 2:** Radiological assessment of the deformity correction [Mean ± SD].

Variable	SM4+2	SP4+2	IM6+2	IP6+2
Patients (n)	53	27	100	50
SCA (°)				
Preoperative	11.3±6.6	12.1±8.2	12.7±9.0	11.7±6.7
Postoperative	1.8±7.1	6.1±6.4	1.5±5.1	2.3±4.4
Correction loss	3.6±2.8	3.8±1.8	1.2±1.1	1.5±1.8
VBA (°)				
Preoperative	16.2±5.7	15.0±5.2	16.1±5.7	15.3±5.5
Postoperative	7.0±5.2	7.5±4.1	7.0±4.6	6.8±3.7
Correction loss	1.4±2.2	1.5±1.9	0.7±1.0	0.8±1.2
AVBH (%)				
Preoperative	65.5±12.5	70.3±8.8	66.7±15.5	68.8±14.0
Postoperative	89.1±11.2	87.4±9.3	96.0±9.7	89.0±10.0
Correction loss	1.8±4.4	3.0±2.3	1.5±1.2	2.3±1.6

**SM4+2/SP4+2**: short-segment monoaxial/polyaxial pedicle screw fixation; **IM6+2/IP6+2**: intermediate monoaxial/polyaxial pedicle screw fixation; **SCA**: sagittal Cobb’s angle; **VBA**: vertebral body angle; **AVBH**: anterior vertebral body height.
